# Prevalence of Risk Factors for Non-Communicable Diseases in the Adult Population of Urban Areas in Kabul City, Afghanistan

**DOI:** 10.5195/cajgh.2013.69

**Published:** 2014-01-03

**Authors:** Khwaja Mir Islam Saeed

**Affiliations:** Afghanistan National Public Health Institute, Ministry of Public Health, Kabul, Afghanistan

**Keywords:** non-communicable diseases, risk factors, hypertension, obesity, WHO STEPS

## Abstract

**Background:**

Non-Communicable diseases (NCDs) are a major global problem. This study aims to estimate the prevalence of common risk factors for NCDs among the adult population in urban areas of Kabul city, Afghanistan.

**Methods and Materials:**

This study was conducted from December 2011 through March 2012 and involved a survey of 1169 respondents, aged 40 years and above. Multistage cluster sampling was used for participant selection, followed by random sampling of the participants. The World Health Organization STEPwise approachfor Surveillance (STEPS) was modified and used for this study.

**Results:**

The overall prevalence of smoking was 5.1% (14.7% men versus 0.3% women) and using mouth snuff was 24.4% in men and 1.3% in women. The prevalence of obesity and hypertension were 19.1% and 45.2 % in men and 37.3% and 46.5% in women. Prevalence of diabetes was 16.1% in men and 12% in women. The overall prevalence of obesity, hypertension and diabetes mellitus was 31.2%, 46% and 13.3%, respectively. On average, subjects consumed 3.37 servings of fruit and 2.96 servings of leafy vegetables per week. Mean walking and sitting hours per week (as proxies for physical activity) were 19.4 and 20.5, respectively. A multivariate model demonstrated that age was a significant risk factor for obesity (OR=1.86), diabetes (OR=2/09) and hypertension (OR=4.1). Obesity was significantly associated with sex (OR=1.65).

**Conclusion:**

These results highlight the need for interventions to reduce and prevent risk factors of non-communicable diseases in urban areas of Kabul City, Afghanistan.

Non-communicable diseases (NCDs) consist of a vast group of non-infectious medical conditions; however emphasis has been on cardiovascular disease, cancer, diabetes, and chronic respiratory diseases. Despite being included in the recent, global development agenda, 1 the World Health Assembly also endorsed an important, new health goal to reduce avoidable mortality from NCDs 25% by 2025. 2 In 1990, there were 26.6 million deaths worldwide from NCDs, which increased to 34.5 million in 2010. 3 Furthermore, in 2008 approximately 63% (36 million) of all deaths were due to NCDs. 4 Likewise, the total number of disability-adjusted life-years (DALYs) increased from 43% in 1990 to 54% in 2010 worldwide. 5 The global economic burden of NCDs is large, estimated at US $6.3 trillion in 2010, anticipated to rise to $13 trillion by 2030. 6

Due to rapid demographic and epidemiologic transitions, South Asian life expectancy is increasing and fertility rate is reducing, which has led to an increased health burden of NCDs. 7 The leading risk factors for the development of non-communicable disease are high blood pressure, high cholesterol, inadequate intake of fruit and vegetables, overweight or obesity, physical inactivity, and tobacco use. 8 It has been indicated that the prevalence of smoking varies in South Asian countries from 16–32%, alcohol consumption (3–41%), eating less than five servings of fruits and vegetables (81–99%), physical inactivity (4–24%), overweight and obesity (9–44%), elevated blood pressure (8–42%), elevated fasting blood sugar (4–9%), and elevated blood cholesterol (13–54%). 9 In a recent Indian study, it was shown that those living in urban areas were at higher risk for developing NCDs as compared to their rural counterparts. 10

Hypertension affects nearly one billion people worldwide and it is expected to increase to 1.5 billion by the year 2025. 11 The global prevalence of hypertension is estimated to be 30% among adults, with variations existing between economically developed and developing countries. 12 In the Eastern Mediterranean Region (EMR), the prevalence of hypertension is approximately 29%, affecting approximately 125 million individuals. 13 In Iran, the estimated prevalence of hypertension in those aged 30–55 and more than 55 years was approximately 23% and 50%, respectively. 14 In a Pakistani study, the overall prevalence of hypertension was 26%, with changes in sex as well as age. 15 Global diabetes prevalence was 2.8% in 2000 and is projected to grow to 4.4% by 2030. 16 Developing countries are increasingly vulnerable to obesity and diseases associated with excess adipose tissue. 17,18 In another Pakistani study, the prevalence of diabetes was 12.1% in males and 9.8% in females, 19 while the national prevalence of diabetes in Iran in 2005 was 7.7% with 8.3% of females and 7.1% of males affected. 20

Complete information of the morbidity and mortality associated with NCDs is not fully available in Afghanistan due to low attention and years of conflict. However, the Afghanistan Mortality Survey (AMS) in 2010 revealed that 33.3% of all deaths are attributed to NCDs compared to 42.6% due to communicable, maternal, perinatal, and nutritional conditions. Cardiovascular diseases, malignant neoplasms, diabetes, respiratory diseases, and digestive diseases are the leading causes of deaths due to NCDs. 21 Based on a World Health Organization (WHO) estimate, in 2000 there were 468,000 people with diabetes in Afghanistan. This number is expected to rise to 1,403,000 by 2030, representing nearly a threefold increase. 22,23 Smoking prevalence among men 15 years and older in Kabul city was reported as 35%, 24 compounded by the fact that Kabul ranked as one of the dirtiest cities in the world in terms of ambient air quality, potentially increasing the burden of respiratory diseases and different types of cancer among humans. 25

The STEPwise approach to Surveillance (STEPS) developed by WHO has been used by different countries in order to identify and monitor the prevalence of NCD risk factors. 26 In the EMR, more than half of member states (56%) have already conducted and established surveillance systems for NCDs using STEPS. 27 Unfortunately, there is no experience of surveillance of NCDs in Afghanistan. This study aims to describe the prevalence and risk factors for NCDs among adults aged 40 years and older in Kabul City, Afghanistan using standardized survey methodology, STEPS, 28 and to provide information for public health actions.

## Methods and Materials

### Setting

This cross-sectional study was conducted among residents 40 years old and above in Kabul city, the capital of Afghanistan. The city has approximately 534,900 households and 3,289,000 inhabitants, with a slightly higher male population (51.8%). 29 Administratively, Kabul city is divided into 22 districts, in 16 of which people reside. Districts are further divided into clusters or neighborhoods (*Gozar*). Initially, we officially requested that the Kabul municipality provide us the list of all neighborhoods, local representative leaders, and its estimated populations; we were able to obtain information on 13 districts.

### Sample size

The statistical software program Epi Info was used to calculate the sample size for this study. By taking into account a precision level of 5%, confidence interval of 95%, and proportion of risk factors in research studies such as diabetes, blood pressure, physical activity, dietary behavior, obesity, age, level of education, and smoking status in diseased and non-diseased people in similar settings, the number of subjects in this study was raised to 600 individuals. To balance considerations of cost, resources, and time without compromising the representativeness of the sample, a three-phase cluster sampling technique was planned. Lastly, after taking into account the design effect (DE=2) of cluster sampling, the final sample size reached (2 × 600) = 1,200, which was reasonable for achieving study objectives within limited resources and funding support.

### Participants

Eligible subjects were selected by multistage sampling; in the first stage, a sample of neighborhoods was selected randomly from each district. In the second stage in each neighborhood a main masjid was selected as a hallmark and heads of households around the masjid were asked to approach the team settled there. In the third stage, one adult person was selected from each household randomly and interviewed after consent was taken. Of our target 1,200 individuals, 1,193 records were available for entry. Seven were lost during fieldwork or transportation. Ten subjects were excluded due to being less than 40 years old, and 14 others were excluded due to unavailability of data for either height or weight for calculation of BMI. Therefore, 1,169 individuals were included in analysis. The study was approved by the Institutional Review Board (IRB) of the Ministry of Public Health, Afghanistan.

### Data Collection and Measurement

Data collection was carried out from December 2011 to March 2012. Data collected by questionnaire consisted of demographic characteristics, socio-economic factors, and behavioral risk factors such as smoking, fruit and vegetable consumption, and physical activity. The questionnaire was modified with expanded and optional questions to suit local needs. Extended questions were in the STEPS instrument modified by adding locally relevant responses. Optional questions were added to the instrument because they were deemed locally important; for example, snuff use was included due to its high prevalence in the area. The questionnaire was translated into the Dari language and tested prior to actual data collection. Physical measurements included weight in bare feet with usual clothing, height in bare feet without headwear, waist circumference at the narrowest point between the lower costal border and the iliac crest measured using a constant tension tape, and blood pressure at the midpoint of the arm after participants had rested for at least five minutes. Two blood pressure readings were obtained for all participants. A third reading was taken if there was a difference of more than 20 mmHg for systolic blood pressure or 10 mmHg for diastolic blood pressure between the first two readings. The mean of all measures was used and recorded. Biochemical measures included random and blood glucose measured in capillary blood using a glucometer. Data collection staff consisted of paramedical doctors and experienced surveyors. They underwent intensive training and supervision provided by the Afghan National Public Health Institute. A pilot study including 20 participants from MoPH staff was conducted, which helped us to reword some questions and estimate the timing of the interview. Blood pressure instruments were tested against each other and showed no differences in measurement. Questionnaires were administered by face-to-face interviews.

### Definitions of risk factors

Overweight was defined as BMI 25–29.9 kg/m^2^, and obesity as BMI ≥30 kg/m^2^. 30 Waist-circumference ≥94 cm in men and ≥80 cm in women was taken as the cutoff point to define central obesity. 31 Consumption of fruits and vegetables were measured as the frequency of servings per week as raw leafy vegetables and one medium-sized and seasonal piece of fruit. Physical activity was defined as walking, sport, or strong physical activity in minutes per day, hours per day, and/or per week. Hypertension was diagnosed if systolic blood pressure was ≥140 mm of Hg and/or diastolic pressure ≥90 mm of Hg, or diagnosed cases taking antihypertensive drugs. 32 Pre-hypertension is defined as 120–139 mm Hg systolic blood pressure and/or 80–89 mm Hg diastolic pressure. 33 Individuals with a random blood sugar of ≥ 200mg/dL were later confirmed by fasting blood sugar (FBS). 34,35 An FBS of ≥ 126mg/dL were considered diabetic. 35

### Statistical Software

Responses in the questionnaire were coded and entered into Epi Info software version 3.5.1. 36 Statistical analyses were performed using SPSS software version 20. 37

## Results

### Descriptive analysis

The mean age of study subjects was 49.5 ± 10.2 years, while according to recent survey in 2010 women and men are now living past age 60 years. 66.5% were female due to low availability of males during daytime at home. The majority of participants (58%) were less than 50 years of age. Illiteracy, being 79.4%, was the main concern in women. Fifty-two percent of the participants had a monthly income of less than 200 USD. Nearly 5% of participants reported being current smokers, and 9% reported using mouth snuff. Reflecting cultural practice, the prevalence of cigarette smoking and mouth snuff used were much higher in men than in women. Selected socio-economic and demographic characteristics of the participants are shown in Table 1.

Two-thirds of families reported using solid ghee for cooking in the kitchen. Fifty percent of participants ate red meat 1–2 times per week while 90% consumed rice 1–2 times per week. Consuming leafy vegetables and fresh fruits with lunch or dinner was less common due to economic problems; close to 60% consumed three servings or less with or after meals. Table 2 presents data on behavioral risk factors for males and females. The adult population, particularly women, in Kabul city performed little physical activity. Approximately 15% of respondents reported 10–30 minutes of different kinds of physical activity daily while the rest reported being sedentary. Walking as a proxy for physical activity was a better habit among citizens. One hour of daily walking for various purposes was quite common (31%) among respondents.

Overall, 69.3% of study participants were either overweight or obese (38.1% overweight and 31.2% obese). Being overweight and obese was slightly more prevalent in women as compared to men. Furthermore, 57.8% were centrally obese using waist circumference. The average BMI was 27.85 ± 5.17 kg/m^2^ and ranged from 15.2 to 62.2 kg/m^2^. 33%, 48.5%, and 18.5% were hypertensive, pre-hypertensive and normotensive, respectively. Table 3 shows the prevalence of pathophysiological risk factors.

The prevalence of diabetes mellitus was 13.3% and was more common in women (59.5%) as compared to men (40.4%). Some study subjects had only one risk factor while the others had two or more, which were categorized as risk factors groups. For instance, having risk factors of diabetes mellitus, blood pressure, and obesity at the same time were categorized as triple risk factor group. Having double risk factors of diabetes and obesity was 5.2%, diabetes and hypertension was 7.8%, and hypertension and obesity was 18.3%. Trip risk factors were present in 3.6% of the participants. The greater the number of risk factors, the higher the probability of NCDs among the adult population.

### Statistical analysis

Multivariate logistic regression was performed for common risk factors versus pathophysiological risk factors such as diabetes mellitus, hypertension and obesity in order to find independent associations,as reflected in Table 4. Age was a significant risk factor for all three conditions with an Adjusted Odds Ratio (AOR) and Confidence Interval (CI) of (AOR=1.86, 95% [1.29, 2.66]), (AOR=2.09, 95% CI [1.32, 3.31], (AOR=4.1, 95% CI [2.97, 5.65]), respectively for obesity, diabetes and hypertension. There were no significant associations between sex and diabetes mellitus and hypertension. However, obesity was significantly associated with sex, with females more likely to be obese as compared to males (OR=1.65, 95% CI [1.08, 2.52]). Illiterate participants were less likely to be diabetic (OR=0.55, 95% CI [0.34, 0.88]) as compared to literate participants.

There were significant associations between diabetes and obesity (OR=1.66, 95% CI [1.04, 2.65]) as well as obesity and hypertension (OR=2.08, 95% CI [1.49, 2.90]). Central obesity was significantly associated with hypertension (OR=1.72, 95%CI [1.25, 2.36]). Proxies for physical activities were daily walking, mode of transportation for getting to the workplace, and sitting time at work or living place. Walking in ‘hours per week’ was significantly associated with obesity, and proxies were associated with hypertension and diabetes mellitus. Diet was categorized by using reported habits of eating red meat or chicken, type of kitchen fat, rice, and fruits and vegetables by weekly or monthly frequencies. Consuming chicken as lunch or dinner was associated with obesity and hypertension while eating red meat as a meal was significantly associated with diabetes mellitus. Cooking meals with liquid ghee was a protective factor for diabetes (OR=0.35, 95% CI [0.22, 0.54]), although we did not find any association of cooking fat with obesity and hypertension. Consuming rice as a meal is considered a luxury in the country. Frequently eating rice as a meal was significantly associated with diabetes (OR=2.3, 95% CI [1.48, 3.58]) and hypertension (OR=1.43, 95% CI [1.06, 1.93]). We found a significant association between frequency of consuming fruits and hypertension (OR=1.43, 95% CI [1.03, 1.96]) but no association between fruits and vegetables with obesity and diabetes mellitus.

## Discussion

Maternal health, malnutrition, vaccine preventable diseases, and communicable diseases are the main concerns in Afghanistan on which the government and its partners are focusing over the last decade. However, epidemiological studies focusing on risk factors for NCDs are of great importance to fill the gap of information and knowledge existing in the country. Our study presented the NCD risk factor burden using WHO-STEPS tool estimated prevalence of diabetes, smoking, snuff use (smokeless tobacco), obesity, blood pressure, and physical inactivity among the urban population of Kabul city. It could be used as a baseline for newly established NCD department at MoPH to develop policies and strategies and plan of actions against them.

According to our findings, two thirds of the adult (age ≥40) urban citizens of Kabul city, the capital of Afghanistan, are overweight or obese with nearly one third of them meeting the criteria for obesity. In addition, more than half of the adult population has central obesity. Although no information is available to assess the trends of obesity, the economic condition of urban citizens is getting better. Moreover, culturally being overweight and obese is perceived as healthy, and people are not interested in losing weight; particularly fat women are considered to be beautiful. Increasing age has a negative effect on the level of obesity at bivariate as well as multivariate levels. Based on recent studies the life expectancy is 62 years in the country. 38 Since the study was conducted on upper age groups, it can be justified that older ages suffering from chronic diseases, which are attributed to low appetite and being on a diet, are not gaining weight. Likewise, gender as a non-modifying factor has a relationship at both level of analysis. As females are mostly confined at home as housewives and are less likely to be physically functional, they are not as obese as compared to males. Therefore, health promotion strategies should focus on obesity as a problem of productive groups. The proportion as well as the effect of age and gender on obesity is comparable with other studies. 39–45

The independent significant association of obesity and blood pressure as well as diabetes depict that the factors are related to each other and combined intervention concentrating on multiple factors is beneficial. With the prevalence of hypertension and diabetes found here, we can posit that the country has already entered an epidemic of non-communicable diseases, which requires strengthening efforts for its control and prevention. The health education campaigns should be tailored to cover all of them. Walking as a proxy for physical activity is a protective factor against obesity as well as other NCDs. Community awareness and establishment of sport centers and jogging lots, which is lacking in urban settings particularly for women, should be encouraged and discussed with relevant sectors. This statement was proved by this study as well as other studies. 19,46,47 Our study shows the higher percentage of blood glucose in people who are equal to or more than 40 years of age (13.57%) in Kabul city. As there is no data regarding prevalence of diabetes in the country, this information can fill the gap of information and help the newly established NDC department at MoPH to plan and focus on target groups. The diabetic control center should be strengthened in the country, and they should focus on older productive age groups with preventive and modifying interventions of life style. Using bivariate analysis, age was a significant non-modifiable factor, which influenced the prevalence of diabetes by change of 9% from lower to higher age groups. High prevalence of diabetes in older age groups who also have other aging diseases will put them in a vicious circle, which could lead to overburdening the health system in the country. Working ahead of time for such diseases, not only reduces the economic burden on health system but also improves the life style of productive groups. Men are at more risk than women, which is not a modifying factor and could be due to genetic makeup. These findings are consistent with national studies in neighboring countries and other nations in the region. 23,48–50

Based on our findings, the prevalence of smoking and snuffing is much higher in men versus women that are probably due to cultural unacceptability and less freedom of smoking among women versus men. As the baseline for smoking is not available it is not supposed to higher. In summary, according to multivariate model we are recommending strategic actions to be concentrated on older ages, education level, cousin marriage to reduce history of diabetes in the family, using liquid oil in kitchen, and short daily walking. Focusing on these factors certainly will lead to reduction of cases in future.

According to the findings of this study, approximately half of the adult population in Kabul city has hypertension. Results of an Iranian study support these findings, in which the overall prevalence rate of hypertension was 27.1% with higher rates in men (32.3%) as compared to women (22.5%). It provides a baseline, or a trigger point, for policy makers to focus upon and take it seriously for future planning and potential interventions. In addition, there was a positive correlation between the hypertension prevalence and age as well as education. The link between education and health is now well established, and growth in literacy rates, particularly among women, has been shown to have a positive impact on health. It seems that non-communicable diseases are occurring as a combined syndrome in adult population. Convenient modes of transportation to the workplace and sedentary lifestyle (including decreased physical activity) have a negative impact on health and the risk of hypertension. The prevalence of diabetes and obesity, either central or general, is affecting the level of blood pressure at the bivariate level of analysis and needs to be considered as comorbidity while managing hypertension. It means that some screening program, if established, will prevent the development of raised blood pressure. Multivariate analysis revealed higher age, education level as socioeconomic factors, consuming chicken, rice, fruits and walking habits as behavioral factors are significantly and independently affecting the level of blood pressure, diabetes and obesity among the adult population in Kabul city. This small study hypothesize that the country is entering in critical situation of NCDs while still no attention is given to them. Other research conducted in Angola and China found similar findings that older age, lower level of education, retirement/unemployment and higher body mass index were significantly associated with hypertension. 51–55 It seems that the factors are mostly associated with each other and/or concurrently present in one individual at the same time. The interventions are needed to target a group of risk factors rather than just one or two factors.

This study, despite of having limitations, has provided useful baseline information for policy development and design of interventions. Inclusion of upper age groups has limited the application of findings to population. In the present study, two thirds of participants were females, which are likely had an influence on the results for women because data were not weighted for age and sex to national population. The under representation of men in this age group was due to some being away from home at the time of the survey. However, the findings of our study illustrated for the first time the prevalence of main NCD risk factors in an urban setting of the country. The main limitation of the study was financial resources for covering cost, which might have affected the result of the study by not going door to door and randomly interviewing individuals or performing glucose tolerance test instead of testing fasting blood sugar. The technique used for recruiting the study participants could encourage participation of those with chronic diseases and leading to possible overestimated prevalence of chronic diseases including obesity. Not withstanding this study demonstrates the impact of non-communicable diseases such as diabetes, blood pressure, and obesity on a population already significantly burdened by communicable and preventable diseases.

Research institutions and the MoPH to develop more rigorous research and national studies to assess the impact of non-communicable diseases in Afghanistan may use these findings. Suffering from non-communicable diseases such as diabetes, high blood pressure, and obesity requires concerted interventions aimed toward prevention. The findings obtained from this study can contribute in formulation of more advanced and national studies to have a generalized picture of non-communicable diseases and their risk factors in the country. It also will assist policy makers to develop a strategy for appropriate control and prevention of non-communicable diseases in the Afghan urban population.

## Figures and Tables

**Table 1:** Frequency distribution of the background characteristics of study participants (N=1,169).

VARIABLES	CATEGORIES	MALE (%)	FEMALE (%)	TOTAL (%)
Age in years (missing values =17)				
	40 – 49	152 (22.2)	532 (77.8)	684 (58.5)
	50 – 59	108 (45.6)	129 (54.4)	237 (20.3)
	60 – 69	88 (55.7)	70 (44.3)	158 (13.7)
	70 and over	39 (53.4)	34 (46.6)	73 (6.3)
Level of education				
	Illiterate	138 (20.6)	531 (79.4)	669 (57.2)
	Primary/Unofficial Education	59 (50.4)	58 (49.6)	17 (10.0)
	Secondary School	116 (54.7)	96 (45.3)	212 (18.2)
	High school and more	79 (46.2)	92 (53.8)	171 (14.6)
Monthly income (Afghanis) (missing=181)				
	≤ 10000	187 (36.1)	331 (63.9)	518 (52.4)
	10000 – 20000	96 (33.0)	195 (67.0)	291 (29.5)
	20000 – 30000	33 (41.8)	46 (58.2)	79 (8.0)
	≥ 30000	53 (53.0)	47 (47.0)	100 (10.1)
Work Status				
	Government Employee	120 (46.7)	137 (53.3)	257 (22.0)
	Business	52 (88.1)	7 (11.9)	59 (5.0)
	Farmer/worker	52 (85.2)	9 (14.8)	61 (5.2)
	Jobless	63 (13.5)	404 (86.5)	467 (39.9)
	Unable to work	37 (94.9)	2 (5.1)	39 (3.3)
	Housewife	68 (23.8)	218 (76.2)	286 (24.5)
Smoking Status (missing=9)				
	Current Smoker	57 (96.6)	2 (3.4)	059 (05.1)
	Ever Smoker	85 (89.5)	10 (10.5)	095 (08.2)
	Never Smoker	246 (24.5)	760 (75.5)	1,006 (86.7)
Mouth Snuff Use Status (missing =10)				
	Current User	95 (90.5)	10 (9.5)	105 (9.0)
	Ever User	23 (92.0)	2 (8.0)	25 (2.2)
	Never User	271 (26.3)	758 (73.7)	1,029 (88.8)

**Table 2:** Frequency distribution of the Behavior factors evaluated (n=1,169).

VARIABLES	CATEGORIES	MALE (%)	FEMALE (%)	TOTAL (%)
Type of kitchen oil				
	Solid Oil 56	264 (32.6)	547 (67.4)	811 (69.4)
	Solid Oil (no)	128 (35.8)	230 (64.2)	358 (30.6)
	Liquid Oil 56	120 (35.8)	215 (64.2)	335 (28.7)
	Liquid Oil (no)	272 (32.6)	562 (67.4)	834 (71.3)
Frequency of eating red meat in a month (missing=8)				
	3 times per month	102 (33.7)	201 (66.3)	303 (26.1)
	3–6 times/month	164 (28.2)	418 (71.8)	582 (50.1)
	6–9 times/month	61 (38.1)	99 (61.9)	160 (13.8)
Frequency of eating rice in a month (missing=88)				
	3 times per month	140 (26.3)	392 (73.7)	532 (49.2)
	3–6 times/month	173 (40.6)	253 (59.4)	426 (39.4)
	>6 times/month	56 (45.5)	67 (54.5)	123 (11.4)
Frequency of taking vegetables per week				
	Once of a week	50 (21.2)	186 (78.8)	236 (20.2)
	Twice a week	81 (28)	208 (72)	289 (24.7)
	Thrice a week	61 (28)	102 (62.6)	163 (13.9)
	More than 3 times a week	200 (41.6)	281 (58.4)	481 (41.1)
Frequency of taking fruits per week				
	Once of a week	59 (33.5)	117 (66.5)	176 (15.1)
	Twice a week	76 (28.7)	189 (71.3)	265 (22.7)
	Thrice a week	77 (33.3)	154 (66.7)	231 (19.8)
	More than 3 times a week	180 (36.2)	317 (63.8)	497 (42.5)
Frequency of physical activity per day in minutes				
	10 minutes per day	319 (31.6)	691 (68.4)	1,010 (86.4)
	10–30 minutes/day	61 (46.6)	70 (53.4)	131 (11.2)
	>30 minutes/day	12 (42.9)	16 (57.1)	28 (02.4)
Frequency of sedentary lifestyle per week in hours				
	10 hours per week	100 (42.6)	135 (57.4)	236 (20.1)
	10–30 hours/week	220 (27.4)	583 (72.6)	804 (68.7)
	>30 hours/week	71 (55.0)	58 (45.0)	129 (11.0)
Frequency of walking per week in hours (missing=7)				
	10 hours per week	133 (35.8)	238 (64.2)	371 (31.9)
	10–30 hours/week	181 (29.2)	439 (70.8)	620 (53.4)
	>30 hours/week	74 (43.3)	97 (56.7)	171 (14.7)

**Table 3:** Frequency distribution of pathophysiological risk factors for NCDs of study participants (N=1,169).

Variables	Categories	Male (%)	Female (%)	Total (%)
Basic Mass index (in kg/m square)				
	Underweight	6 (46.2)	7 (53.8)	13 (1.1)
	Normal weight	144 (41.6)	202 (58.4)	346 (29.6)
	Overweight	167 (37.5)	278 (62.5)	445 (38.1)
	Obese	75 (20.5)	290 (79.5)	365 (31.2)
Central Obesity (missing=14)				
	Yes	234 (48.9)	245 (51.1)	479 (41.0)
	No	155 (22.9)	521 (77.1)	676 (57.8)
Blood Pressure				
	Normotensive	181 (31.9)	386 (68.1)	567 (48.5)
	Pre-hypertensive	105 (49.1)	109 (50.9)	214 (18.3)
	Hypertensive	106 (27.3)	282 (72.7)	388 (33.2)
Diabetes Mellitus				
	No	329 (32.5)	684 (67.5)	1,013 (86.7)
	Yes	63 (40.4)	93 (59.6)	156 (13.3)
Diabetes Mellitus and Obesity				
	Both Factors	18 (29.5)	43 (70.5)	61 (5.2)
	One Factors	102 (25.6)	297 (74.4)	399 (34.1)
	No Factors	272 (38.4)	437 (61.6)	709 (60.7)
Diabetes Mellitus and Hypertension				
	Both Factors	36 (39.6)	55 (60.4)	91 (7.8)
	One Factors	168 (32.8)	344 (67.2)	512 (43.8)
	No Factors	188 (33.2)	378 (66.8)	566 (48.4)
Hypertension and Obesity				
	Both Factors	36 (16.8)	178 (83.2)	214 (18.3)
	One Factors	180 (37.9)	295 (62.1)	475 (40.6)
	No Factors	176 (36.7)	304 (63.3)	480 (41.1)
Hypertension, Obesity and Diabetes Mellitus				
	All 3 Factors	10 (23.8)	32 (76.2)	42 (3.6)
	One or 2 Factors	225 (32.5)	468 (67.5)	693 (59.3)
	No Factors	157 (36.2)	277 (63.8)	434 (37.1)

**Table 4:** Multivariate analysis risk factors associated with obesity, hypertension, and diabetes mellitus.

	OBESITY			DIABETES MELLITUS			HYPERTENSION		
Variables	*AOR (95% CI)*		*p-value*	*A OR (95% CI)*		*p-value*	*AOR (95% CI)*		*p-value*
Age group									
<50 years	1	Reference	-	1	Reference	-	1	Reference	-
>50 years	1.86	1.29 – 2.66	<0.01	2.09	1.32 – 3.31	<0.01	4.10	2.97 – 5.65	<0.001
Sex									
Male	1	Reference	-	1	Reference	-	1	Reference	-
Female	1.65	1.08 – 2.52	<0.01	0.88	0.52 – 1.47	>0.05	0.93	0.63 – 1.35	>0.05
Education Level									
Literate	1	Reference	-	1	Reference	-	1	Reference	-
Illiterate	1.24	0.86 – 1.80	>0.05	0.55	0.34 – 0.88	<0.01	1.35	0.97 – 1.88	>0.05
Diabetes Mellitus									
No	1	Reference	-	-	-	-	1	Reference	-
Yes	1.66	1.04 – 2.65	<0.05	-	-	-	1.19	0.76 – 1.86	>0.05
Blood Pressure									
No	1	Reference	-	1	Reference	-	-	-	-
Yes	2.08	1.49 – 2.90	<0.05	0.82	0.52 – 1.28	>0.05	-	-	-
Obesity ( BMI>30)									
No	-	-	-	1	Reference	-	1	Reference	-
Yes	-	-	-	1.64	1.02 – 2.63	>0.05	2.16	1.55 – 3.01	<0.001
Central Obesity									
No	1	Reference	-	1	Reference	-	1	Reference	-
Yes	5.45	3.76 – 7.89	<0.001	1.18	0.73 – 1.88	>0.05	1.72	1.25 – 2.36	<0.001
Walking habit per week									
>30 hours	1	Reference	-	1	Reference	-	1	Reference	-
<30 hours	0.81	1.10 – 2.99	<0.05	1.37	0.70 – 2.70	>0.05	1.33	0.86 – 2.04	>0.05
Sitting 10 hours per week									
<10 hours	1	Reference	-	1	Reference	-	1	Reference	-
>10 hours	1.12	0.74 – 1.70	>0.05	3.52	1.78 – 6.96	<0.001	1.13	0.86 – 2.04	>0.05
Mode of transportation (using car for going to work)									
No	1	Reference	-	1	Reference	-	1	Reference	-
Yes	0.86	0.42 – 1.76	>0.05	2.16	1.04 – 4.48	<0.01	1.17	0.59 – 2.28	>0.05
Using Chicken as meal									
<3 times per month	1	Reference	-	1	Reference	-	1	Reference	-
>3 times per month	0.62	0.46 – 0.88	<0.01	0.80	0.52 – 1.22	>0.05	1.36	1.01 – 1.83	<0.05
Using Liquid Ghee in Kitchen									
No	1	Reference	-	1	Reference	-	1	Reference	-
Yes	1.08	0.75 – 1.56	>0.05	0.35	0.22 – 0.54	<0.001	1.04	0.74 – 1.45	>0.05
Red meat 3 times per month									
<3 times per month	1	Reference	-	1	Reference	-	1	Reference	-
>3 times per month	1.04	0.72 – 1.50	>0.05	1.81	1.14 – 2.87	<0.05	1.05	0.75 – 1.45	>0.05
Rice 3 times per week									
<3 times per week	1	Reference	-	1	Reference	-	1	Reference	-
>3 times per week	0.85	0.65 – 1.19	>0.05	2.30	1.48 – 3.58	<0.001	1.43	1.06 – 1.93	<0.05
Fruits twice a week									
<twice a week	1	Reference	-	1	Reference	-	1	Reference	-
>twice a week	0.92	0.65 – 1.30	>0.05	0.62	0.38 – 1.02	>0.05	1.43	1.03 – 1.96	<0.05
Vegetable once a week									
<once a week	1	Reference	-	1	Reference	-	1	Reference	-
>once a week	0.95	0.62 – 1.44	>0.05	0.85	0.45 – 1.56	>0.05	1.28	0.87 – 1.88	>0.05
Smoking status									
No	1	Reference	-	1	Reference	-	1	Reference	-
Yes	1.66	1.04 – 2.65	<0.05	2.32	0.97 – 5.50	>0.05	1.13	0.55 – 2.33	>0.05
